# Fresh Pork Quality Assessment by NIRS and NMR: Predicting Eating Quality and Elucidating Relationships with Key Chemical Components

**DOI:** 10.3390/ani15202973

**Published:** 2025-10-14

**Authors:** Xiying Li, Melindee Hastie, Minh Ha, Robyn D. Warner, Cameron C. Steel, Peter McGilchrist, Evan McCarney, Darryl N. D’Souza, Robert J. E. Hewitt, David W. Pethick, Maddison T. Corlett, Sarah M. Stewart, Frank R. Dunshea

**Affiliations:** 1 School of Agriculture, Food and Ecosystem Sciences, Faculty of Science, The University of Melbourne, Parkville, VIC 3010, Australia; lixiying@zqu.edu.cn (X.L.); robyn.warner@unimelb.edu.au (R.D.W.); 2 School of Food and Pharmaceutical Engineering, Zhaoqing University, Zhaoqing 526061, China; 3 v2food, Melbourne, VIC 3121, Australia; 4 School of Environmental and Rural Science, University of New England, Armidale, NSW 2350, Australia; 5 inMR Measure Ltd., Wellington 6012, New Zealand; 6 SunPork Group, Eagle Farm, Brisbane, QLD 4009, Australiarobert.hewitt@sunporkfarms.com.au (R.J.E.H.); 7 School of Veterinary and Life Sciences, Murdoch University, Murdoch, Perth, WA 6150, Australia; 8 Faculty of Biological Sciences, University of Leeds, Leeds LS2 9JT, UK

**Keywords:** collagen, intramuscular fat, pH, sensory evaluation, correlation, near-infrared spectroscopy (NIRS), nuclear magnetic resonance (NMR)

## Abstract

The Australian pork industry has been seeking a rapid and non-destructive way to predict pork chemical components and eating quality. In this study, near-infrared spectroscopy (NIRS) and nuclear magnetic resonance (NMR) were applied to fresh pork *Longissimus thoracis et lumborum* (LTL) and *Semimembranosus* (SM) with the aim to build prediction models for intramuscular fat (IMF) content, collagen content and solubility, pH, and sensory attributes, namely tenderness, juiciness, liking of flavor and overall liking as well as investigate the effects of chemical components on pork eating quality. Results showed that the NIRS output, which was a predicted IMF content calibrated for the IMF of lamb, correlated with the chemically analyzed IMF content across both muscles. In LTL, NMR parameter *p_2f_* was weakly correlated with IMF and pH. For the LTL, NMR parameters *p_21_* and *p_22_* were related to sensory tenderness, while *T_22_* was correlated with the liking of flavor. In both muscles, the collagen content and pH were related to all sensory attributes, and IMF was related to the liking of flavor. The chemical properties of SM were weakly correlated with those of LTL. The NIRS and NMR weakly predicted the pork chemical components and sensory properties, but more studies are required to improve the accuracy.

## Simple Summary

Near infrared spectroscopy (NIRS) and nuclear magnetic resonance (NMR) are non-destructive and rapid ways to predict the physiochemical properties and eating quality of pork. The results of this study showed that NIRS and NMR were able to predict the intramuscular fat content of pork loin and topside, but their accuracy was still low. Some NMR parameters could be used to predict the eating quality of pork, but again, further studies are required to improve the accuracy of the results.

## 1. Introduction

The Australian pork industry has been seeking rapid and non-destructive methods to measure the chemical components and eating quality of pork. Near-infrared spectroscopy (NIRS) and nuclear magnetic resonance (NMR) are promising techniques to replace destructive chemical assays and predict the meat-eating quality as they are rapid and cost-effective.

NIRS measures the absorption of electromagnetic radiation at wavelengths of 750–2500 nm, which correspond to the overtones and vibrations of chemical bonds [1]. Previously, NIRS has been reported to predict the pH, color and color stability, drip loss, intramuscular fat (IMF) content, collagen characteristics, and sensory properties of pork [2,3].

NMR spectroscopy is widely used to determine the molecular structure, while proton relaxometry also provides information on the behavior of water, which is abundant in meat [4]. NMR relaxometry can be used to predict the water-holding capacity, IMF content, pH and color, and sensory properties [5,6]. However, there are still some technical limitations for both NIRS and NMR, and online applications in the pork industry are difficult. Errors in the model may exist, and food safety issues, detection limits, references, and the implementation of equipment in the pork industry need further research [7].

Attempts have been made to predict the eating quality of pork using its chemical components including collagen and IMF. Wheeler et al. [8] built a prediction model for tenderness using the collagen content, sarcomere length, and desmin degradation, and the collagen content was negatively related to tenderness with a slope of −0.46 ± 0.047. Fortin et al. [9] found that IMF had linear relationships with sensory chewiness (slope = −0.51) and flavor intensity (slope = 0.18). However, the effects of collagen and IMF on pork eating quality are debatable, as several studies found that they had little effect on the pork eating quality [10]. A meta-analysis conducted by Li et al. [11] reported that collagen content and solubility had a significant relationship with sensory tenderness in the model for beef, but the data for pork was insufficient to conduct a similar analysis.

The loin muscle *Longissimus thoracis et lumborum* (LTL) is often used to indicate carcass quality and has been extensively studied [12]. However, chemical components and eating quality differ between muscles. A system that can effectively indicate the quality of other primals or carcasses based on the measurement of loin will be beneficial for the pork industry. Huff-Lonergan et al. [13] reported that the pH of pork *semimembranosus* (SM) was correlated with the pH of LTL. Font-i-Furnols et al. [14] found that the IMF of pork SM, *biceps femoris* (BF), and *gluteus medius* (GM) were correlated with the IMF of LTL. Arkfeld et al. [12] suggested that the pH and color traits of pork LTL were correlated with those of pork ham, but the Pearson’s r values were low. Limited studies have been conducted on the correlations in collagen characteristics between pork LTL and SM.

Therefore, we hypothesize that:

- NIRS and NMR parameters could be used to predict the IMF, collagen characteristics, pH, and sensory attributes of pork;
- Chemical measurements could be used to predict the eating quality of pork;
- Chemical measurements of SM could be predicted by LTL measurements.

This study aimed to:

- Study the difference in the IMF, collagen characteristics, pH, and sensory properties between pork LTL and SM;
- Build prediction models for the IMF, collagen characteristics, pH, and sensory attributes of pork using NIRS and NMR parameters;
- Investigate the effects of chemical measurements on pork eating quality;
- Investigate whether chemical measurements of the two pork muscles were correlated.

## 2. Materials and Methods

### 2.1. Sample Preparation

Samples were collected from a commercial abattoir (SunPork Group, Kingaroy, Qld, Australia) from 60 randomly selected commercial carcasses graded to a heavy female carcass market. These carcasses had an average hot standard carcass weight of 101.6 ± 1.49 kg (mean ± SE), and a fat depth at the P2 site (65 mm from the midline at the last rib on the left) of 14.4 ± 0.33 mm as measured by ultrasonic scanning (AutoFom, Frontmatec Group, Kolding, Denmark). Slaughter occurred according to normal commercial conditions, with the group of pigs stunned utilizing a back-loading carbon dioxide paternoster stunner (90% CO_2_, 3.5 min) prior to exsanguination and entering a vertical steam scald tunnel (60 °C, 10 min transit time). Carcasses were dressed prior to splitting along the backbone and removal of the skull, with halves remaining on a common gambrel. Pigs left the kill floor after 43 min and entered a quick-chill tunnel for 106 min (entry temperature −20 °C; exit temperature −15 °C) before entering equalization chillers (3 °C, 40% fan speed) until boning the following day. Sample collection occurred on a single day in April under a scavenged tissues collection approval (Ethics ID 22013) from the University of Melbourne Large Animal Ethics Committee. At 24 h post-mortem, LTL and SM with skin were excised from each carcass. Each muscle was cut in the middle without cutting through the skin. A hand-held near-infrared spectroscopy (NIRS) device was applied to the cross-section area at three points. The details of the NIRS device are described below. The pH and temperature were measured at the same site. After finishing all the measurements, the muscles were vacuum packed, frozen at ~48 h post-mortem, and transported to the University of Melbourne. Samples were kept frozen at −20 °C until further analysis.

All muscles were cut frozen using a meat and bone bench band saw (CARNIVORE equipment, Melbourne, VIC, Australia). From each LTL, a 4 cm long steak for nuclear magnetic resonance (NMR) and the chemical assays was cut out starting from the cutting line at the middle of the muscle toward the posterior end. For NMR, the steak was vacuum-packed and frozen (see below for the measurement details). From the anterior side of the cutting line, a 12.5 cm long trunk was cut out, vacuum packed, and kept frozen until ready for sensory evaluation. From each SM, a 2 cm long steak from the cutting line was cut out for the chemical assays. Muscles of the other part of the cutting line were vacuum packed and stored frozen for sensory evaluation. The SM was not subjected to NMR measurement. Samples for the chemical assays were freeze-dried (ZIRBUS VaCo10, ZIRBUS technology GmbH, Bad Grund, Germany) for 48 h, powdered by knife, and kept frozen (−20 °C) until analysis.

### 2.2. pH Measurement

The pH and temperature were measured on the cross-section of the muscle using a portable pH/temperature meter (TPS WP-80M, Brendale, QLD, Australia). The pH probe was calibrated with pH buffers 4.01 and 7.01, and the temperature probe was calibrated using one-point calibration at 0 °C.

### 2.3. Near-Infrared Spectroscopy (NIRS)

The portable NIRS device (S-7090, SOMA Optics Ltd., Tokyo, Japan) had a lithium-ion battery as a power supply and a tungsten lamp as its light source, all contained within a hard waterproof external shell made of ABS resin. The portable NIR device uses the wavelength range from 885 to 1015 nm and was calibrated using the partial least squares regression method on the IMF content of the LTL from 1318 lambs extracted using chloroform in a Soxhlet (R^2^ = 0.77, RMSECV = 0.75).

Prior to measurement of the pork samples, the NIR device was internally calibrated using a white Duracon^®^ resin disk that was housed in the device lens cap. After the loin muscle was cut ~24 h post-mortem, the lens of the NIR device was placed in contact with the cut surface of the LTL and SM. The predicted IMF value was shown on the screen of the NIRS device. Three measurements were taken for each muscle, removing the device and repositioning it at another site on the loin between scans. The mean, geometric mean, and the highest value of the three measurements were calculated for further analysis.

### 2.4. Nuclear Magnetic Resonance (NMR) Relaxometry

Steaks retained for NMR relaxometry were thawed at 4 °C in the vacuum-packed pouch, placed on the NMR surface, and measured sealed. The same sample was measured three times by flipping and adjusting the position of the muscle.

The NMR measurements were made on a Marbl™ flatbed NMR device from inMR Measure Ltd. (Wellington, New Zealand). This instrument is a unilateral NMR system operating at 5.1 MHz that was developed and optimized to measure intramuscular fat in chilled meat. The NMR design is similar to that described in McCarney et al. [15], with a sweet spot sensitive region between the surface and 2 cm into the sample, 2 cm wide and about 5 cm long.

Transverse relaxation was measured using the Carr–Purcell–Meiboom–Gill sequence (CPMG) [16,17]. A total of 2000 echoes were collected with an echo spacing of 0.3 ms, and a polarization delay of 750 ms was observed at the end of each scan. The NMR response was signal averaged until the signal to noise ratio was greater than 200, which is a compromise between an acceptable acquisition time and acceptable measurement error.

The points collected within each echo were averaged and fit to a tri-exponential equation:

(1) $$I=\;\sum^{i}p_{2i}e^{-\frac{t}{T_{2i}}}$$

where *I* is the signal intensity, *p_2i_* is the exponential amplitude, *T_2i_* is the relaxation time, and subscript *i* designates the population components f, 1, or 2. *T_2f_* was fixed at 10 ms to stabilize the fitting algorithm, and the five other variables, *p_2f_*, *p_21_*, *p_22_*, *T_21_*, and *T_22_* were obtained. The means of the three measurements of the same sample were used for analysis. *P_2f_* represents the amplitude of the short exponential decay and is related to water associated with macromolecules. At cool temperatures, the relaxation rate of fat decreases [18], and the linear coefficient *p_2f_* correlates with intramuscular fat in red meat [19]. *T_21_* and *T_22_* are the two characteristic transverse relaxation time constants that are associated with water within the myofibrillar proteins and inter-myofibrillar water, respectively [20]. *P_21_* and *p_22_* are the two corresponding amplitudes that are described by their respective time constants.

### 2.5. Chemical Assays

Intramuscular fat (IMF) content was determined by AOAC method 991.36 [21] following the description of Li et al. [22] with some modifications. Duplicates of 3.5 g freeze-dried pork powder were wrapped in folded Watman no.1 filter paper and subjected to Soxhlet extraction. The extraction solvent was diethyl ether, and the extraction lasted for 2 h. The IMF content was measured gravimetrically and expressed as the percentage of IMF of fresh pork.

Collagen content and solubility were determined using AOAC method 990.26 [23] for the determination of hydroxyproline content colorimetrically, as described previously by Li et al. [22]. Collagen content was expressed as mg collagen per g of fresh pork. Collagen solubility was expressed as the percentage of soluble collagen over the total collagen content.

### 2.6. Sensory Evaluation

#### 2.6.1. Consumers

Sensory evaluation of this project was approved by the University of Melbourne Human Ethics Committee (Ethics ID: 2022-24524-32998-4). One hundred and ninety-eight untrained consumers were recruited who were all above 18 years old, had consumed pork in the past three months, were willing to consume pork, and did not consume any coffee or strong-tasting food for at least one hour prior to the test. All consumers provided informed consent via the statement “I consent to participate in this project, the details of which have been explained to me, and I have been provided with a written plain language statement to keep”. Sensory evaluation was conducted over three days with three to four sessions per day. There were 18 to 20 consumers in each session.

Sensory evaluation followed the methods described by Channon et al. [24] with some modifications. All consumers attended a briefing and filled out a demographics questionnaire before tasting commenced. Each consumer was given seven samples to assess. The first serving was a Link sample that was mid quality LTL from a previous project from the same supplier. The Link sample was used for consumers to familiarize themselves with the sensory assessment questionnaires and establish a common sensory baseline. Consumers filled in their responses to the questionnaires on the tablets provided (Samsung Galaxy View, Samsung Electronics Co. Ltd., Suwon, South Korea) and were given plain crackers and 10% apple juice to cleanse their palate between samples.

#### 2.6.2. Sensory Sessions

All muscles were randomly assigned across the three days of sensory sessions with equal numbers of SM and LTL assigned to each day. Samples were thawed at 0–2 °C for 24 h. All subcutaneous fat and connective tissues were removed. These were cut across the muscle fiber into steaks that were 4.0 cm × 4.0 cm with a 2.5 cm thickness, with five steaks prepared from each muscle. All steaks were randomized within day and across sessions. The order of consumption was arranged in a 6 × 6 Latin square so that each sample was consumed before and after each other to avoid sample order bias. These were 50% vacuum packed (50% of air was taken out), with labels printed on sheets of laminated A4 paper. All samples were stored at 0–2 °C before cooking and prepared one to two days before cooking.

On the cooking day, samples for specific sessions were placed in a Styrofoam box and transported to the kitchen 15 min before the session started. The steaks were cooked in a Silex grill set at 160 °C on both sides. There were ten steaks on the grill at the same time. These were grilled to an internal temperature of 68 °C, rested for 30 s, cut in half, and served. The final internal temperature was around 72 °C. A set of starter samples and a thermocouple were used to determine the cooking time for the day. The cooking time was 3 min 45 s to 4 min. Ten servings were obtained from each muscle (with each steak cut in half after cooking). Each muscle was tasted by ten consumers.

#### 2.6.3. Questionnaires

The demographic questionnaire was a printed copy (Supplementary Materials). Questions included gender, age group, cultural heritage, number of people in the household, parent or guardian of children under the age of 18, occupation of the main income earner of the household, household yearly income, and pork consumption frequency. These were multiple-choice questions where the consumers could only select one answer. The demographic information of the consumers is shown in Table S1.

The tasting questionnaire (Supplementary Materials) was modified from Channon et al. [24]. The questionnaire was loaded onto a website using RedJade Sensory Software (RedJade Sensory Solutions, LLC, Martinez, CA, USA). Tenderness, juiciness, liking of flavor, and overall liking were on continuous line scales, and the words on two ends of the line scales were: tenderness—0 (not tender) and 100 (very tender); juiciness—0 (not juicy) and 100 (very juicy); liking of flavor—0 (dislike extremely) and 100 (like extremely); overall liking—0 (dislike extremely) and 100 (like extremely).

In addition, there were three multiple-choice questions. The first question was “Do you detect any off-flavor?” and the answer was “yes” or “no”. The second question was about purchase intent, and the answers were: 1—I would definitely not buy it; 2—I would probably not buy it; 3—I might buy it; 4—I would probably buy it; 5—I would definitely buy it. The third question was about quality grading and the answers were: 1—Unsatisfactory; 2—Good everyday; 3—Better than good everyday; 4—Premium.

A check-all-that-apply (CATA) question formed the last part of each assessment, where the consumers selected the terms that best described the sample they tasted. The CATA terms used included 15 flavor terms: “bitter”, “buttery”, “clean”, “earthy”, “fecal”, “familiar”, “fatty”, “metallic”, “porky”, “roasted”, “sweet”, “sour”, “salty”, “savory (umami)”, and “tasteless”, and 6 texture terms: “chewy”, “dry”, “fibrous”, “juicy”, “soft”, and “tender”.

### 2.7. Data Analysis

Chemical data were analyzed by a restricted maximum likelihood (REML) generalized linear model in GenStat (22nd edition, VSN International, Hemel Hempstead, UK). The fixed factor was muscle. For line scale sensory data, these were analyzed with the generalized linear mixed-effect model (GLME) in RStudio Version 2024.12.1 (RStudio, PBC, Boston, MA, USA) using packages “lme4”, “emmeans”, and “jtools”. The fixed model was muscle, and the random model was cooking_day/participant + carcass.

For purchase intent and quality grading, success was defined as the consumers selecting 5 (I definitely would buy it) and 4 (I would probably buy it) for purchase intent as well as 4 (Premium) and 3 (Better than good everyday) for quality grading. The probability of no off-flavor and the probability of success were calculated by the GLME model in RStudio with logarithmic transformations and binomial distribution. The fixed model was muscle, and the random model was cooking_day/participant + carcass. The probability of being selected for a CATA term was analyzed with the same method.

The effects of chemical measurements on pork eating quality were analyzed with the GLME model in RStudio with the fixed model as muscle + IMF + collagen content + collagen solubility + pH and the random model as session/participant + carcass.

The relationship of chemical measurements between LTL and SM was analyzed by a simple linear model in RStudio with measurements of SM as y and measurements of LTL as x.

The predictions of chemical measurements by the NIRS output and NMR parameters were first tested by correlation matrices. Then, those with the highest Pearson’s r were selected and visualized in plots with the regression equation and R^2^. The prediction of sensory attributes was calculated by the GLEM model with a fixed model as the NIRS output (+ muscle) or NMR parameter and a random model as participant/session + carcass. Root mean squared errors (RMSEs) were calculated for each model.

## 3. Results

The SM had a higher pH (*p* < 0.001), lower collagen solubility (*p* = 0.004), and higher IMF content (*p* < 0.001) than the LTL (Table 1). IMF ranged from 0.443% to 2.25% in LTL and from 0.579% to 2.84% in SM. There was no difference in collagen content between the two muscles. The SM also had a higher sensory tenderness score (*p* = 0.014), juiciness score (*p* < 0.001), and overall liking score (*p* = 0.010, Table 2).

The liking of flavor score tended to be higher in the SM than in LTL (*p* = 0.063, Table 2). The probability of no off-flavor was higher in the LTL than in SM (*p* = 0.013). However, no difference was found between the two muscles for the probability of success for purchase intent and quality grading. The results of the selected CATA terms showed that LTL had more selections for “tasteless”, “dry”, “sour”, and “fibrous” than SM, while SM had more selections for “metallic”, “juicy”, “familiar”, “porky”, and “tender” than LTL (Table S1).

Table 3 details the effects of chemical measurements on the sensory attributes of pork across both muscles. IMF was positively related to the liking of flavor (*p* = 0.034). Collagen content was negatively related to tenderness (*p* = 0.002), juiciness (*p* = 0.010), liking of flavor (*p* = 0.008), and overall liking (*p* = 0.001). Collagen solubility was negatively related to juiciness (*p* = 0.039). pH was positively related to tenderness (*p* = 0.029), juiciness (*p* = 0.023), liking of flavor (*p* = 0.022), and overall liking (*p* = 0.011).

The pH of SM was related to the pH of LTL (R^2^ = 0.273, *p* < 0.001), as shown in Table 4. The collagen content and collagen solubility of SM were also predicted by those of LTL (R^2^ = 0.214, *p* < 0.001 for collagen content; R^2^ = 0.101, *p* = 0.012 for collagen solubility). However, the IMF content of the two muscles was not significantly related.

**Figure 1:**
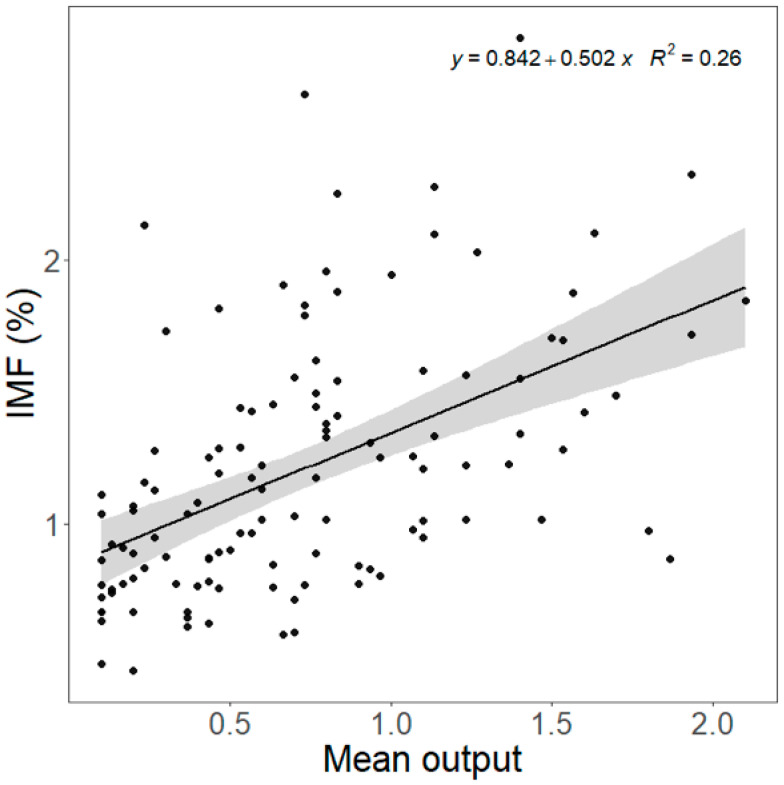
Prediction of chemically analyzed intramuscular fat (IMF) content by mean near-infrared spectroscopy output of *Longissimus thoracis et lumborum* (LTL) and *Semimembranosus* (SM). Slope = 0.502 ± 0.079, R^2^ = 0.258, *p* < 0.001, RMSE = 0.413. Shaded area is the 95% confidence interval.

**Figure 2:**
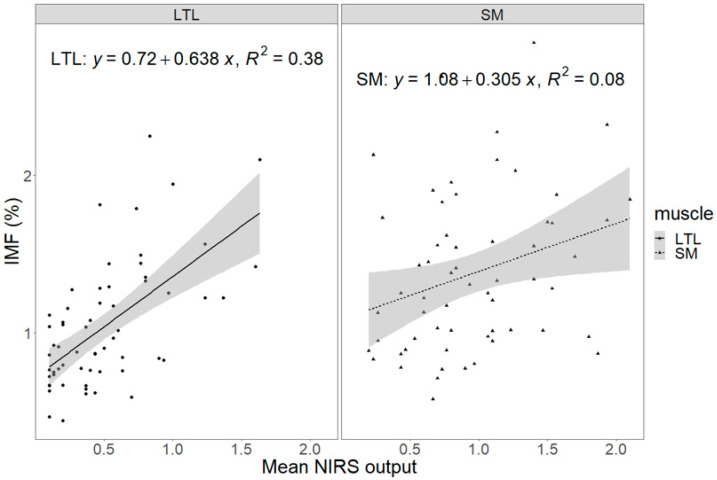
Prediction of chemically analyzed intramuscular fat (IMF) content by mean near-infrared spectroscopy output of *Longissimus thoracis et lumborum* (LTL) and *Semimembranosus* (SM). LTL: slope = 0.638 ± 0.108, R^2^ = 0.375, *p* < 0.001, RMSE = 0.311; SM: slope = 0.305 ± 0.108, R^2^ = 0.083, *p* = 0.025, RMSE = 0.477. Shaded area is the 95% confidence interval.

The mean, geometric mean, and highest NIRS output were correlated with chemically analyzed IMF content (Table S2), and the highest R^2^ was with the mean output (R^2^ = 0.258, *p* < 0.001, Figure 1). The mean NIRS output was positively related to chemically analyzed IMF content in both the LTL (R^2^ = 0.375, *p* < 0.001) and SM (R^2^ = 0.083, *p* = 0.025). However, the slope, R^2^, and *p* values were higher in LTL, and the RMSE was lower in LTL (Figure 2). For the relationship between NIRS output and sensory attributes, no significant relationship was found in LTL, SM, or for both muscles (Table 5).

**Figure 3:**
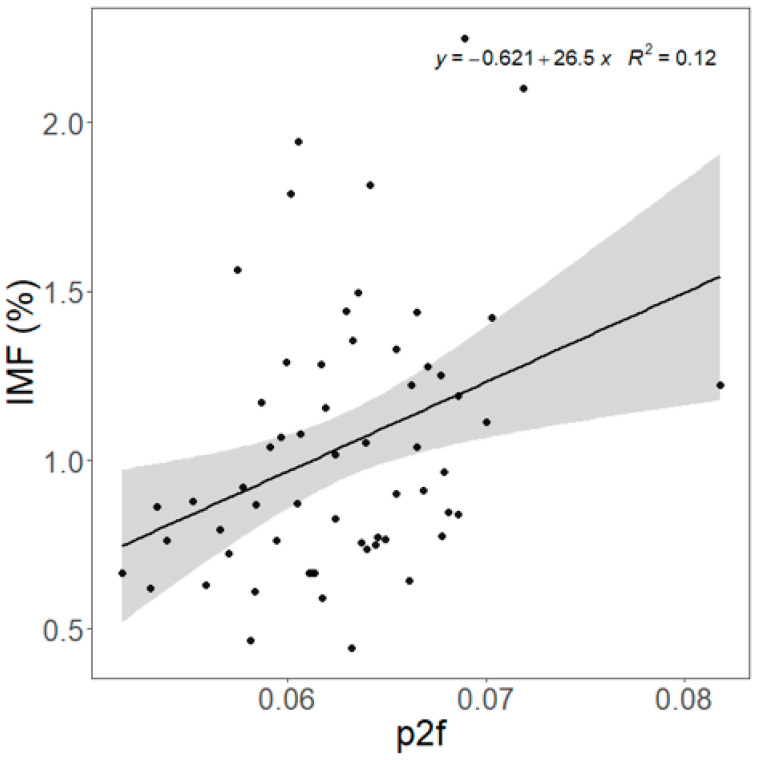
Prediction of the chemically analyzed intramuscular fat (IMF) content of the *Longissimus thoracis et lumborum* (LTL) by the nuclear magnetic resonance parameter *p_2f_* value. Slope = 26.5 ± 9.22, R^2^ = 0.124, *p* = 0.006, RMSE = 0.366. Shaded area is the 95% confidence interval.

**Figure 4:**
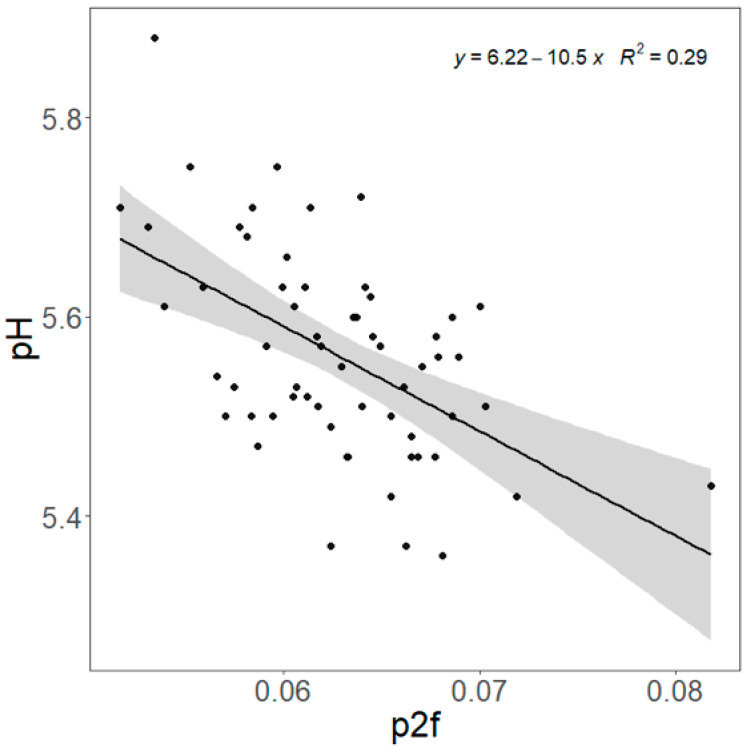
Prediction of pH of the *Longissimus thoracis et lumborum* (LTL) by nuclear magnetic resonance parameter *p_2f_* value. Slope = −10.5 ± 2.17, R^2^ = 0.287, *p* < 0.001, RMSE = 0.094. Shaded area is the 95% confidence interval.

NMR parameter *p_2f_* was correlated with chemically analyzed IMF (R^2^ = 0.124, *p* = 0.006, Figure 3) and pH (R^2^ = 0.287, *p* < 0.001, Figure 4). *P_2f_*, *T_21_*, and *T_22_* were correlated (*p* < 0.05) with pH (Table S3). For sensory attributes, *p_21_* was positively related to tenderness (*p* = 0.007, Table 6), while *p_22_* was negatively related to tenderness (*p* = 0.008). *T_22_* was negatively related to liking of flavor (*p* = 0.010). The relationship between *T_22_* and tenderness was close to significant (*p* = 0.056). However, if random terms were removed, the relationships between *T_22_* and tenderness became significant (*p* = 0.010, Table S4). Also, significant relationships were found between *T_22_* and liking of flavor as well as between *p_21_*, *p_22_*, and overall liking. The NIRS outputs were positively correlated (*p* < 0.05) with *p_2f_* and negatively with *T_21_* (Table S5).

## 4. Discussion

The major findings of this study were that (1) NIRS outputs and NMR parameters were significantly but weakly correlated with IMF content in LTL; (2) NIRS outputs could not predict sensory attributes, while NMR parameters *p_21_* and *p_22_* were related to tenderness, and *T_22_* was related to the liking of flavor; (3) IMF, collagen content, and pH were related to some sensory attributes (tenderness, juiciness, liking of flavor, and overall liking); and (4) chemical measurements of LTL were not good indicators for those of the SM. Therefore, hypotheses (1) and (2) were partly accepted, and hypothesis (3) was rejected.

The application of NIRS in predicting the chemical components, physical measurements, and sensory properties of meat have been extensively studied [25]. In commercial practices, the FOSS FoodScan™ near-infrared spectrophotometer (FOSS A/S, Hillerød, Denmark) with an artificial neural network calibration model has become an AOAC official method for fat, moisture, and protein [26]. In previous studies, the R^2^ for calibration ranged from 0.35 to 0.76 for intact pork [27,28,29,30]. However, in the present study, the SOMA NIRS output was weakly correlated with the chemically analyzed IMF with an R^2^ of 0.38 in LTL and 0.08 in SM, although the slopes were significant. The greater variation in the SM was not unexpected because of the more heterogeneous nature and size of this muscle. This device was originally calibrated for lamb loin. The range of IMF that the SOMA is AUS-MEAT approved for is from 3.5 to 8% [31], which is much higher than that of the pork in this study. Also, the range in pork IMF content assessed in this study was limited and overall quite low compared with other pork studies conducted outside Australia, where the IMF content was 2.7 ± 1.3% for LTL [28]. We also suggest that the sampling area was small for heterogeneous muscles such as LTL and SM [30]. Despite the very low concentrations of IMF, there were significant relationships between the predicted IMF and actual IMF. While these values are unlikely to be good enough to provide confidence in predicting the IMF within the low ranges of IMF encountered in Australian pork LTL and SM, they do provide encouragement that with some finessing of the instrumentation and algorithms, which are specifically developed for pork, these will soon be overcome.

The NMR parameters were weakly correlated with the IMF content and pH in pork LTL in the present study. Pooke and McCarney [19] demonstrated that the NMR parameters, especially *p_2f_*, were correlated with the IMF in lamb with the highest R^2^ at 0.70. However, Brown et al. [32] reported that the R^2^ between the IMF and NMR signals was 0.073, while Brøndum et al. [28] reported an R^2^ of 0.46. In the present study, there were some high outliers in the chemical estimates of IMF content, and these values did appear to be outliers in a number of relationships. However, we have no reason to doubt these values, and removing the three highest values did not improve any of the relationships. The NMR parameters mainly reflect the water properties in the meat [33], and the pH can affect water properties [34]. Therefore, the NMR parameters are correlated with pH. However, the number of samples and range of chemical measurements were small. More samples with a wider range may result in better prediction. In addition, NMR was applied on freeze-thawed pork in this study. Freeze-thawing has been found to affect the meat water-holding capacity and *T_2_* characteristics [35], which may influence the accuracy of the models. If the models can be improved, the measurements on freeze-thawed pork may be used in the analysis of the quality of exported or imported frozen pork, while NIRS is more suitable for use in the abattoir.

The prediction of sensory attributes by NMR parameters has been of great interest to the meat industry, but a solid relationship is lacking. Bertram et al. [36] found that *T_2_* data was correlated with juiciness (r = 0.82–0.87) and tenderness (r = 0.86). Similar results were reported by Fjelkner-Modig and Tornberg [37], although a majority of NMR parameters were not significantly correlated with the sensory attributes. In the present study, *p_21_* had a positive relationship with tenderness, while *p_22_* had a negative relationship. Water acts as a plasticizer in meat, and more water within the myofibril can increase meat tenderness [38]. On the other hand, a greater amount of extracellular water results in greater drip loss and cooking loss, which reduces tenderness [37]. As *p_21_* is related to intra-myofibrillar water and *p_22_* is related to inter-myofibrillar water, they are related to pork tenderness. The lack of a significant relationship between juiciness and NMR parameters could be due to differences in the statistical model, the subjectivity of consumers, and the highly variable nature of questionnaires. Also, muscles in the present study were freeze-thawed, which could impact the water-holding capacity of the muscles and the NMR relaxation parameters. Future studies on fresh pork muscles need to be conducted.

In the present study, the collagen content was negatively related to all sensory attributes. However, there were contradictory findings on the effects of collagen content on meat tenderness in previous studies. Some authors found that the collagen content was negatively correlated with sensory tenderness [39,40,41], while others reported that the collagen content had little effect on sensory tenderness [42,43,44]. In a previous meta-analysis, the collagen content significantly affected beef tenderness, but the relationship was weak and varied among muscles [11]. It is possible that collagen affects sensory tenderness, but the characteristics of collagen, such as collagen solubility and cross-links as well as other muscle components, also matter [45,46]. The synergetic effects of muscle components contribute to the meat-eating quality. Also, these studies varied in animal factors, such as leanness, age and sex and slaughter weight, which might contribute to the variation between studies in the contribution of collagen to tenderness. In the present study, the IMF content was low, and the effects of collagen might become significant.

Aside from the collagen content, the pH was also positively related to all sensory attributes in this study. The positive effect of pH on sensory tenderness and the juiciness of meat has been reported [13,47,48]. The contribution of ultimate pH to meat tenderness is partly due to its effect on enzyme activity, which affects the degradation of myofibrillar proteins during proteolysis [49]. pH also affects the water-holding capacity of raw and cooked meat. When the pH of meat is higher than the isoelectric point of the major proteins, there is repulsion between groups of proteins, leading to greater space to hold water [34]. In addition, the denaturation temperature of myosin and actin is pH-dependent, resulting in less shrinkage and lower cooking loss at higher pH [38]. The positive relationship between pH and flavor could be caused by the sour taste of lower-pH meat, which is disliked by many consumers [50]. Therefore, pH positively contributes to pork tenderness, juiciness, and flavor, which then affects the overall liking.

The IMF content of pork affected the liking of flavor, which was similar to the results reported in the literature [51,52]. Hundreds of volatile compounds, especially those responsible for the characteristic flavor of pork, are derived from fat [53]. Therefore, pork with a higher IMF content will be richer in flavor. Unlike several previous studies, IMF did not influence sensory tenderness or juiciness in this study [54,55]. This is likely caused by the low IMF content in Australian pork, as Barton-Gade and Bejerholm [56] found that a minimum of 2% in IMF was required to have a noticeable influence on sensory attributes. Also, the range of IMF content was small in the present study, resulting in greater variation in the prediction.

The SM had high tenderness and was juicier than the LTL. These findings differed from those reported in the literature, where the LTL received higher tenderness scores or showed lower Warner–Bratzler shear force (WBSF) [8,57]. The lower tenderness of SM is usually attributed to its higher collagen content [58,59]. However, the collagen content did not differ between LTL and SM in the present study. Also, the higher juiciness in SM could be due to its higher IMF content and lower cooking loss than LTL [60], which increases juiciness perception [61]. Pork SM was considered more flavorful than LTL, as shown in the results of CATA, although they did not differ in liking of flavor. These differences contributed to the difference in overall liking between muscles.

Although the pH, collagen content, and solubility of SM were correlated with those of LTL, the R^2^ was low. Similarly, Arkfeld et al. [12] reported that the pH of SM was correlated with that of LTL, but the Pearson’s r was 0.33. Knecht et al. [62] also found that sensory attributes of pork ham primal were not correlated with those of pork LTL. Pork quality traits are complex and can be influenced by various factors, leading to difficulties in predicting the quality of one muscle from another [13]. In addition, the gene expression profile differs between LTL and SM in pigs [63]. As a result, the prediction of pork SM quality from that of LTL had low accuracy.

## 5. Conclusions

Both the NIRS and NMR measurements appeared to be weakly related to pork IMF, particularly in the LTL. These relationships existed despite the very low levels of observed IMF content. pH was positively related to all sensory attributes, while collagen content was negatively related to all attributes. The IMF positively affected the liking of flavor. The chemical properties of LTL were correlated with the pH, collagen content, and solubility of SM, but the correlation was weak, likely because the sample size was small. This technology will help the Australian pork industry implement the online rapid detection of chemical components and eating quality for quality control and grading. It is recommended that pork carcasses be manipulated nutritionally and genetically to increase the range in IMF to improve the eating quality. Further tests of both NIRS and NMR over a greater range in IMF content are required to improve the prediction model.

## Figures and Tables

**Table 1:** Chemical properties of pork *Longissimus thoracis et lumborum* (LTL) and *Semimembranosus* (SM). Values are mean ± SEM (range).

	LTL (*n* = 60)	SM (*n* = 60)	*p*-Value ^1^
pH	5.56 ± 0.014 (5.33–5.88)	5.65 ± 0.018 (5.46–5.99)	<0.001
Collagen content (mg/g)	4.56 ± 0.073 (3.51–5.57)	4.73 ± 0.108 (3.21–8.51)	0.20
Collagen solubility (%)	10.2 ± 0.258 (6.13–18.5)	9.19 ± 0.239 (5.78–13.4)	0.004
IMF ^2^ (%)	1.04 ± 0.051 (0.443–2.25)	1.38 ± 0.065 (0.579–2.84)	<0.001

^1^ Data were analyzed by the generalized linear mixed model. Fixed term = muscle. ^2^ IMF = intramuscular fat.

**Table 2:** Ratings of the sensory properties of pork *Longissimus thoracis et lumborum* (LTL) and *Semimembranosus* (SM).

	LTL (*n* = 593)	SM (*n* = 595)	*p*-Value ^1^
Sensory Score (1–100)			
Tenderness	48.8 ± 2.26	51.7 ± 2.26	0.014
Juiciness	48.1 ± 2.42	54.1 ± 2.42	<0.001
Flavor	52.0 ± 1.73	54.0 ± 1.73	0.063
Overall liking	52.0 ± 1.73	54.7 ± 1.73	0.010
Consumer Probability			
No off-flavor	0.996 ± 0.004	0.992 ± 0.007	0.013
Purchase intent ^2^	0.298 ± 0.031	0.351 ± 0.033	0.093
Quality grading ^3^	0.284 ± 0.030	0.318 ± 0.032	0.27

^1^ Data were analyzed by generalized linear mixed-effects models. Fixed model = muscle; random model = session/participant + carcass. Off-flavor, purchase intent, and quality grading analysis underwent log transformation and binomial distribution. Data were expressed as the mean ± standard error of the mean. ^2^ Success = consumers selected 5 (I definitely would buy it) and 4 (I would probably buy it). ^3^ Success = consumers selected 4 (Premium) and 3 (Better than good everyday).

**Table 3:** Effects of chemical components on pork eating quality in both *Longissimus thoracis et lumborum* (LTL) and *Semimembranosus* (SM).

	Tenderness		Juiciness		Liking of Flavor		Overall Liking	
	Slope ^1^	*p*-Value	Slope	*p*-Value	Slope	*p*-Value	Slope	*p*-Value
pH	14.1 ± 6.41	**0.029 ^3^**	12.6 ± 5.49	**0.023**	11.7 ± 5.05	**0.022**	14.2 ± 5.53	**0.011**
Collagen content (mg/g)	−3.86 ± 1.21	**0.002**	−2.76 ± 1.05	**0.010**	−2.60 ± 0.97	**0.008**	−3.42 ± 1.05	**0.001**
Collagen solubility (%)	−0.79 ± 0.45	0.083	−0.83 ± 0.04	**0.039**	−0.68 ± 0.37	0.065	−0.69 ± 0.39	0.082
IMF ^2^ (%)	0.40 ±1.68	0.81	0.88 ± 1.48	0.55	2.91 ± 1.36	**0.034**	1.58 ± 1.46	0.28

^1^ Data were analyzed by generalized linear mixed effects models. Fixed model = muscle + IMF + collagen content + collagen solubility + pH; random model = session/participant + carcass. Data were expressed as the mean ± standard error of the mean. ^2^ IMF = intramuscular fat. ^3^ Bold number indicated significant results.

**Table 4:** Prediction of chemical components in *Semimembranosus* (SM) from *Longissimus thoracis et lumborum* (LTL).

	Slope ^1^	R^2^	*p*-Value
pH	0.397 ± 0.085	0.273	<0.001
Collagen content (mg/g)	0.312 ± 0.079	0.214	<0.001
Collagen solubility (%)	0.348 ± 0.134	0.104	0.012
IMF (%)	0.165 ± 0.101	0.044	0.11

^1^ Slope was expressed as the mean ± standard error of the mean. Data were analyzed by the linear model, where the dependent variable was the chemical measurements of SM and independent variable was the chemical measurements of LTL.

**Table 5:** Regression coefficients and the *p*-value of the prediction models of sensory attributes by mean and the highest NIRS output of *Longissimus thoracis et lumborum* (LTL) and *Semimembranosus* (SM).

Muscle	Tenderness		Juiciness		Liking of Flavor		Overall Liking	
	Slope ^1^	*p*-Value	Slope	*p*-Value	Slope	*p*-Value	Slope	*p*-Value
Both	−1.60 ± 1.76	0.36	−0.36 ± 1.55	0.81	0.42 ± 1.44	0.77	0.07 ± 1.53	0.96
LTL	1.11 ± 3.07	0.72	1.00 ± 2.69	0.71	−0.47 ± 2.64	0.86	−0.35 ± 2.82	0.90
SM	−2.47 ± 2.33	0.29	−1.99 ± 1.98	0.32	0.38 ± 1.90	0.84	−0.44 ± 2.17	0.84

^1^ Data were analyzed by generalized linear mixed effects models. Fixed model = muscle + NIRS output for both muscles and NIRS output for the individual muscle; random model = participant/session + carcass. Data were expressed as the mean ± standard error of the mean.

**Table 6:** Regression coefficients and *p*-values of the prediction of sensory attributes by nuclear magnetic resonance parameters.

	Tenderness		Juiciness		Liking of Flavor		Overall Liking	
	Slope ^1^	*p*-Value	Slope	*p*-Value	Slope	*p*-Value	Slope	*p*-Value
*p_2f_*	−252 ± 217.0	0.25	−245 ± 190.5	0.20	−323 ± 183.5	0.083	−363 ± 195.4	0.068
*p_21_*	87.1 ± 31.23	**0.007 ^3^**	41.1 ± 28.69	0.16	38.6 ± 28.20	0.18	54.8 ± 29.70	0.070
*p_22_*	−92.0 ± 33.25	**0.008**	−40.2 ± 30.61	0.20	−35.4 ± 30.13	0.25	52.7 ± 31.77	0.10
*T_21_* (ms)	0.80 ± 0.78	0.31	−0.12 ± 0.69	0.86	0.91 ± 0.67	0.18	1.16 ± 0.71	0.11
*T_22_* (ms)	−0.33 ± 0.17	0.056	−0.26 ± 0.15	0.078	−0.37 ± 0.14	**0.010**	−0.21 ± 0.16	0.18

^1^ Data were analyzed by generalized linear mixed effects models. Fixed model = NMR parameter, random model = participant/session + carcass. Data were expressed as the mean ± standard error of the mean. ^3^ Bold number indicated significant results.

## Data Availability

The original contributions presented in this study are included in the article/supplementary material. Further inquiries can be directed to the corresponding author(s).

## Supplementary Materials

The following supporting information can be downloaded at: https://www.mdpi.com/article/10.3390/ani15202973/s1, Table S1: Probability of selected for check-all-that-apply (CATA) terms of pork *Longissimus thoracis et lumborum* (LTL) and *Semimembranosus* (SM). Table S2: Correlation matrices between NIR output and chemical measurements of both muscles. Table S3: Correlation matrices between NMR parameters and chemical measurements. Table S4: Regression coefficients and *p*-value of the prediction of sensory attributes by NMR parameters (without random terms). Table S5: Correlation matrices between NIR output and NMR parameters. DEMOGRAPHICS QUESTIONNAIRE. QUESTIONNAIRE.
