# Supplementary material for: Fresh Pork Quality Assessment by NIRS and NMR: Predicting Eating Quality and Elucidating Relationships with Key Chemical Components
Source: Animals (Basel). 2025 Oct 14;15(20):2973. doi: 10.3390/ani15202973 (PMC12560931; doi:10.3390/ani15202973)
Supplement: Supplementary file 1 [file animals-15-02973-s001.zip › animals-3853052-supplementary.pdf]

Table S1. Probability of selected for check-all-that-apply (CATA) terms of pork *Longissimus thoracis et lumborum* (LTL) and *Semimembranosus* (SM)

	LTL	SM	*P*-value[1]
Number of servings	593	595	
Tasteless	0.168 ± 0.0217	0.0838 ± 0.0144	**<0.001**
Dry	0.324 ± 0.0322	0.204 ± 0.0255	**<0.001**
Sour	0.0892 ± 0.0190	0.0536 ± 0.0131	**0.006**
Metallic	0.0551 ± 0.0112	0.115 ± 0.0180	**<0.001**
Fibrous	0.367 ± 0.0485	0.303 ± 0.0446	**0.044**
Juicy	0.346 ± 0.0364	0.492 ± 0.0396	**<0.001**
Familiar	0.168 ± 0.0233	0.220 ± 0.0271	**0.038**
Porky	0.349 ± 0.0372	0.557 ± 0.0399	**<0.001**
Tender	0.243 ± 0.0237	0.297 ± 0.0260	**0.047**

[1] Data was analyzed by generalized linear mixed effects models. Fixed model = muscle; random model = session/participant + carcass. Data underwent log transformation and binomial distribution. Data was expressed as mean ± standard error of mean.

Table S2. Correlation matrices between NIR output and chemical measurements of both muscles

	Geometric Mean output	Mean output	Highest output
IMF (%)	0.49***	0.51***	0.49***
pH	-0.17	-0.16	-0.13
Collagen content (mg/g)	0.15	0.13	0.07
Collagen solubility	-0.02	0	0.05

Table S3. Correlation matrices between NMR parameters and chemical measurements

	p2f	p21	p22	T21 (ms)	T22 (ms)
IMF (%)	0.35**	-0.1	0.04	-0.11	-0.11
Collagen content (mg/g)	0.07	0.06	-0.08	0.07	-0.04
Collagen solubility	0.2	-0.07	0.04	-0.08	0.19
pH	-0.54***	0.21	-0.13	0.45***	-0.30*

Table S4. Regression coefficients and *P*-value of prediction of sensory attributes by NMR parameters (without random terms)

	Tenderness		Juiciness		Liking of flavor		Overall liking	
	Slope[1]	*P*-value	Slope	*P*-value	Slope	*P*-value	Slope	*P*-value
p2f	-210 ± 191.1	0.27	-200 ± 190.1	0.29	-213 ± 172.5	0.22	-203 ± 181.8	0.27
p21	92.9 ± 28.48	**0.001**	48.4 ± 28.50	0.090	49.4 ± 25.86	0.057	69.2 ± 27.18	**0.011**
p22	-99.6 ± 30.27	**0.001**	-49.6 ± 30.31	0.10	-50.4 ± 27.49	0.067	-73.0 ± 28.89	**0.012**
T21 (ms)	0.564 ± 0.676	0.41	-0.079 ± 0.672	0.91	0.616 ± 0.610	0.31	0.582 ± 0.643	0.37
T22 (ms)	-0.384 ± 0.149	**0.010**	-0.293 ± 0.149	0.045	-0.357 ± 0.135	**0.008**	-0.230 ± 0.142	0.11

[1] Data was analyzed by linear models. Fixed model = NMR parameter. Data was expressed as mean ± standard error of mean.

Table S5. Correlation matrices between NIR output and NMR parameters

	Geo Mean output	Mean output	Highest output
p2f	0.48***	0.44***	0.35**
p21	-0.11	-0.08	-0.07
p22	0.04	0.02	0.01
T21 (ms)	-0.34**	-0.34**	-0.31*
T22 (ms)	-0.05	-0.06	-0.08

DEMOGRAPHICS QUESTIONNAIRE

This information is confidential and will be used for classification purposes only.

D1. Please indicate your gender. (Select one)

☐	☐	☐
Male	Female	Other
[1]	[2]	[3]

D2. In which of the following age groups do you belong? (Select one)

☐	☐	☐	☐	☐	☐	☐
20 or younger	21–30	31–40	41–50	51–60	61–70	71-80
[1]	[2]	[3]	[4]	[5]	[6]	[7]

D3. What is your cultural heritage? (Select one)

[1] ☐ Australian
[2] ☐ Indigenous Australian
[3] ☐ British
[4] ☐ European
[5] ☐ Asian
[6] ☐ African
[7] ☐ South American
[8] ☐ North American
[9] ☐ Other. Please specify:____

D4. Including yourself, how many people are living in your household? This includes infants but does not include students living away from home. (Select one)

☐	☐	☐	☐	☐	☐	☐
1	2	3	4	5	6	7 or more

D5. Are you the parent or guardian of any children age 18 or younger living in your household? (Select one)

☐	☐
Yes	No
[1]	[2]

D6. What's the occupation of the main income earner in your household? (Select one)

[1] ☐ Manager
[2] ☐ Professionals (included health professional etc.)
[3] ☐ Technicians and Trade Workers
[4] ☐ Community and Personal Services Workers
[5] ☐ Clerical and Administrative Workers
[6] ☐ Sales Workers (includes retail sales etc.)
[7] ☐ Machinery Operators and Drivers
[8] ☐ Labourers
[9] ☐ Home Duties
[10] ☐ Student
[11] ☐ Other

D7. Which one of the following ranges includes your total yearly household income, before taxes? (Select one)

☐	☐	☐	☐	☐	☐
Under $25,000 per year	$25,000– $50,000 per year	$50,001– $75,000 per year	$75,001– $100,000 per year	$100,001– $125,000 per year	Over $125,000 per year
[1]	[2]	[3]	[4]	[5]	[6]

D8. How often do you consume pork? (Select one)

[1] ☐ Everyday
[2] ☐ 4-5 times a week
[3] ☐ 2-3 times a week
[4] ☐ Weekly
[5] ☐ Fortnightly
[6] ☐ Monthly
[7] ☐ Less than monthly

Sample No. ______ **Participant code_______**

Follow the test instructions closely. You will be provided with 7 individual samples which all require a test. Each sample will have two portions.

Please inspect, smell and then eat one portion the sample provided. Once you have finished fill out the questions below in section (1).

When you have completed section (1), eat the second portion, then select all attributes that apply in section (2). Then immediately alert the researcher to provide the next sample.

(1)

Tenderness

Not Tender — Very Tender

Juiciness

Not Juicy — Very Juicy

Liking of flavor

Dislike Extremely — Like Extremely

Overall Liking

Dislike Extremely — Like Extremely

Do you detect any off-flavor?

Yes [ ] No [ ]
[1] [2]

To what extent would you purchase this product?

I definitely would not buy it [1] ☐
I would probably not buy it [2] ☐
I might buy it [3] ☐
I would probably buy it [4] ☐
I would definitely buy it [5] ☐

Please tick one of the following to

Rate the quality of the pork sample you have just eaten
Choose **one** only (you must make a choice).
Unsatisfactory [1] ☐
Good everyday quality [2] ☐
Better than everyday quality [3] ☐
Premium quality [4] ☐

(2) Please check the words or phrases which best describe the pork sample you have just tried.

Faecal	☐	Fatty	☐	Sweet	☐
Roasted	☐	Tasteless	☐	Dry	☐
Soft	☐	Sour	☐	Metallic	☐
Chewy	☐	Fibrous	☐	Salty	☐
Juicy	☐	Bitter	☐	Savoury (Umami)	☐
Familiar	☐	Clean	☐	Buttery	☐
Porky	☐	Earthy	☐	Tender	☐
